# Introduction of New and Underutilized Vaccines: Sustaining Access, Disease Control, and Infrastructure Development

**DOI:** 10.1371/journal.pmed.0020286

**Published:** 2005-09-13

**Authors:** Jon Kim Andrus, John Fitzsimmons

## Abstract

For vaccines to reach the greatest number of people requires access, accelerated regional disease control, and the development of public health infrastructure.

This is the second of two articles in the October 2005 issue on ways to create practical markets for vaccines.

Thinking of ways to create practical markets for vaccines poses some interesting challenges, particularly in the context of introducing new and underutilized vaccines. In a complex, ever-changing world, making vaccines, whether they are new or old, available to children and families who need them most should be a top priority [1]. Models for creating markets have been recently developed that might benefit from the experience gained from the regional and national immunization approaches used in the Americas. From the perspective of the Pan American Health Organization (PAHO), strategies to optimize the market and, ultimately, the use of vaccines include three important, overriding guiding principles: access, accelerated regional disease control, and the development of a public-health infrastructure.

## Access Is Essential

Access to preventive and other health services covers several factors, including access to health units and hospitals, trained staff providing quality services, cost-effective technologies, the best use of available technologies, and information to improve the community's knowledge base and best practices.

Inherently, improved access also addresses issues of equity. Equity is a critical cross-cutting concept that drives much of the work of PAHO and member countries. For vaccines, the challenge has always been, and will continue to be, to ensure that all communities benefit from the potential impact of these technologies [2].

## The Example of Rubella

The initiative to eliminate rubella and congenital rubella syndrome in the Americas serves as an example of PAHO's member countries seizing the opportunity to ensure access, while promoting the ultimate goal of disease control (virtual elimination of a disease), and the improvement of the public-health infrastructure [3]. These efforts help ensure that all families receive the benefit of vaccine technology because disease elimination requires that all communities be reached, regardless of ethnic background, religion, or income. Although the rubella vaccine was only recently introduced in many countries of the Americas, many of them simultaneously accelerated the rubella disease control strategies by strengthening surveillance and conducting mass vaccination campaigns to rapidly reduce the pool of rubella-susceptible individuals in the community. Such efforts eventually led to the adoption of a regional rubella-elimination initiative in the Americas by the year 2010 [4].

Public-sector capacity development creates regional independence.

## Targeting High-Risk Districts

In the Americas, targeting high-risk districts with low immunization coverage for special attention also promotes improved access and accelerated disease control. Efforts to strengthen leadership, management, and supervision of program activities have been essential for the success of this strategy. Districts that have less than 95% coverage are targeted for special training, outreach, and follow-up. This targeting helps ensure that even in middle-income countries the substantial disparities that exist in health are addressed. To that end, PAHO's member countries have conducted the annual Vaccination Week in the Americas, during which all countries attempt to reach and vaccinate marginalized, poor populations [5].

## Global Approaches to Vaccination

In the context of access and equity, there is concern about global approaches to support only countries with average annual per capita income of less than $1,000, such as the approach taken by the Global Alliance for Vaccines and Immunization, thus limiting the scope of work and benefits that impoverished children and families could otherwise receive in the Americas. Currently, only six countries are eligible to receive support from the Global Alliance for Vaccines and Immunization: Bolivia, Cuba, Guyana, Haiti, Honduras, and Nicaragua—representing only 7% of the population of the Caribbean and Latin America. However, 11% of people in the Caribbean and Latin America live below the international poverty line ($365 annual per capita income) [6]. In addition, huge discrepancies exist within countries, and these countries urgently need extra resources to ensure access of vaccines to marginalized populations.

## Disease Control in the Americas

In the Americas, efforts to control disease over the last 20 years have led to significant improvements in the public-health infrastructure, particularly in the area of program management, surveillance, and public-health laboratories. In some countries, such as Brazil, the development of public-sector capacity for vaccine production was also a top priority. Such public-sector capacity development creates regional independence, competition to reduce vaccine prices among private-sector producers, and dependable supply chains that are sustained and that contribute to intercountry cooperation [7]. Currently, Brazil produces a yellow fever vaccine that has been used in (and in some cases donated to) neighboring countries suffering from deadly yellow fever outbreaks, for example, the yellow fever outbreak in Colombia in 2004.

## Future Approaches to Practical Markets for Vaccines 

Future approaches that attempt to enhance practical markets for vaccines and that enhance the introduction of new and underutilized vaccines should consider prioritizing the following: access and equity for as much of the population as is possible, well-implemented accelerated disease-control and prevention strategies, and development of a public-health infrastructure.

The general approach also requires high-level political commitment, adequate attention to management, supervision, and logistics, and sound technical strategies. The opportunity to address widespread disparities in health that exist in low- and low-middle-income countries that otherwise do not benefit from Global Alliance for Vaccines and Immunization's marketing approaches will be a huge challenge for the future. PAHO will continue to make the support of immunization to member countries a priority.
